# Graft-versus-host disease-like erythroderma: a sign of recurrent thymoma: A case report

**DOI:** 10.1097/MD.0000000000008877

**Published:** 2017-12-08

**Authors:** Xiujuan Gui, Xinhai Zhu, Liangjun Guo, Guoqiang Tan, Yan Liu, Yi Tan, Qiufang Chen, Yuwei Song, Shaoqiang Lin

**Affiliations:** a Department of Oncology, The First Affiliated Hospital of Jinan University; b Department of Traditional Chinese Medicine, The First Affiliated Hospital of Jinan University; c Department of Central Laboratory, The First Affiliated Hospital of Jinan University; d Department of Clinical Medicine, The First Affiliated Hospital of Guangdong Pharmaceutical University, Guangzhou, China.

**Keywords:** graft-versus-host disease-like erythroderma, pathologic mechanism, thymoma, thymoma-associated multiorgan autoimmunity

## Abstract

**Rationale:**

Thymomas are associated with numerous autoimmune disorders, such as myasthenia gravis (MG), pure red cell aplasia (PRCA), and systemic lupus erythematosus (SLE). However, graft-versus-host disease (GVHD)-like erythroderma is a relatively uncommon paraneoplastic disorder associated with thymomas and signifies a poor prognosis.

**Patient concerns:**

A 35-year-old woman with medical history significant for stage IVa type AB thymoma presented with patchy erythema over face, trunk, and extremities that failed to respond to topical steroids.

**Diagnosis:**

A contrast-enhanced computerized tomography (CECT) scan of the chest demonstrated tumors in the right mediastinum and right pleura. Percutaneous right mediastinal pleural biopsy confirmed recurrent thymoma (WHO type B3, Masaoka stage IVb). Histopathologic examination of her skin lesions revealed GVHD-like erythroderma.

**Interventions:**

The patient received chemotherapy and local thoracic radiotherapy, as well as corticosteroids.

**Outcomes:**

The eruptions gradually subsided with hyperpigmentation; however the patient eventually died of multiple organ failure.

**Lessons:**

GVHD-like erythroderma is an uncommon paraneoplastic disorder associated with thymomas. Though its pathogenesis still needs further research, prompt diagnosis and appropriate treatment can improve survival rate in patients.

## 1 Introduction

The thymus is the most important central immune organ where functional T-cells mature and immune tolerance is induced. Thymomas originate from the epithelial cells of the thymus, and they account for 50% of anterior mediastinum tumors. In addition, they are associated with various autoimmune disorders, such as myasthenia gravis (MG), pure red cell aplasia (PRCA), and systemic lupus erythematosus (SLE). Graft-versus-host disease (GVHD)-like erythroderma is a relatively uncommon paraneoplastic disorder associated with thymomas. GVHD is typically a major complication following allogeneic stem cell marrow transplantation, but it also occurs after blood transfusions or solid organ transplantations that usually involve the skin, liver, and gastrointestinal tract. Thymoma-associated GVHD was first described by Kornacki^[1]^ in 1995; however, thymoma-associated GVHD has rarely been reported in the English literature and the pathogenesis is not completely understood.^[2]^ We describe a patient with a recurrent thymoma who presented with an unusual form of erythroderma that histopathologically resembled GVHD.

## 2 Case report

A 35-year-old woman sought evaluation at a local hospital in February 2016 for scaly papules with pruritus involving the extremities. She was initially diagnosed with eczema, and steroid ointment was prescribed for the cutaneous lesions. However, topical steroids failed to have an effect. The scattered, patchy rashes gradually extended from her extremities to her trunk, and were associated with severe pruritus. The patient also reported lethargy and weight loss, and was subsequently admitted to our hospital in July 2016. The medical history was significant for a thymoma (WHO Type AB, Masaoka stage IVa) diagnosed 9 years earlier, for which she underwent an extended thymectomy, followed by adjuvant radiotherapy and chemotherapy with cyclophosphamide, Pharmorubicin, and cisplatin. She achieved complete remission at that time, and was thereafter examined at regular intervals without evidence of thymoma recurrence.

**Figure 1:**
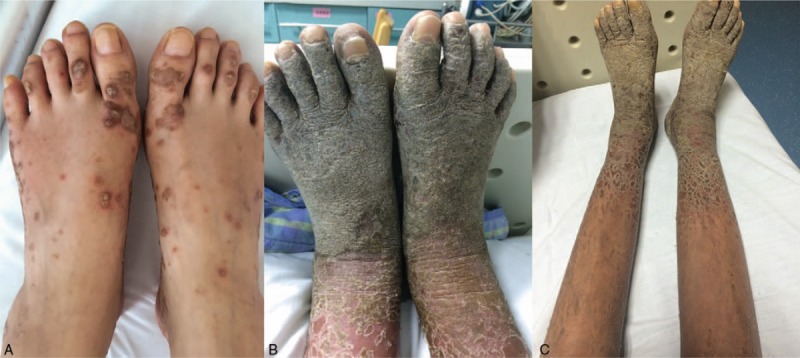
(A) Mutiple aubergine, scaly papules coalesced into patches throughout the body.(B) Scattered patchy rash merged with patchy erythema and severe scaling with acropodium keratinization.(C) Eruptions gradually subsided with hyperpigmentation after treatment.

Physical examination on this admission revealed purple, scaly papules that coalesced into patches on the face, trunk, and upper and lower extremities (Fig. 1A). There were also oral ulcerations and abdominal distension with shifting dullness, but no jaundice. Laboratory testing revealed a normal white blood cell count, normal liver and renal function testing, as well as negative serologic tests for several infectious pathogens (*Treponema pallidum*, hepatitis virus B, hepatitis virus C, and human immunodeficiency virus). The serum levels of CA-125 (234.17 μ/mL; range 0–35) and CA15–3 (53.7 μ/mL; range 0–31.3) were elevated. A contrast-enhanced computerized tomography (CECT) scan of the chest and epigastrium demonstrated tumors in the right mediastinum and right pleura, and metastatic tumors in the liver and lungs. Bone scintigraphy revealed active lesions in the pubic bone bilaterally and left side of the seventh costal cartilage. The patient underwent a CT-guided right mediastinal pleural biopsy which demonstrated a type B3 thymoma, thus categorizing her at stage IVb disease per WHO classification.

**Figure 2:**
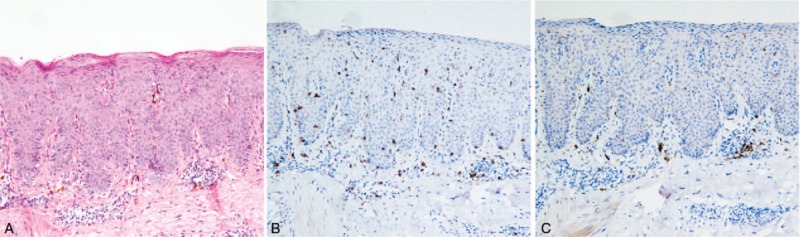
(A) Parakeratosis, liquefaction, and apoptosis of keratotic cells in the epidermis, and the dermis shows perivascular lymphocytic infiltration (hematoxylin-eosin [HE], original magnification ×100). (B) CD8 staining showing CD8+ cells significant infiltrating into the epidermis (×100). (C) CD1a staining shows CD1a+ Langerhans cells nearly disappearing from the epidermis (×100).

The histopathologic examination of a biopsy specimen from the skin lesion on the right thigh revealed parakeratosis, acanthosis, spongiotic, and necrotic keratinocytes throughout the epidermal layer, and perivascular lymphocytic infiltration in the superficial dermis (Fig. 2A). Immunohistochemical staining revealed epidermotropic infiltrations of T-cells (CD8+ > CD4+) (Fig. 2B) and the number of CD1a+ Langerhans cells was significantly reduced in the epidermis (Fig. 2C). These pathologic alterations were similar to those seen in GVHD.^[3]^ Interestingly, this patient never underwent allogeneic stem cell marrow transplantation, blood transfusion, or solid-organ transplantation. Given the presence of GVHD-like skin symptoms and recurrent thymoma, the diagnosis of thymoma-associated GVHD-like erythroderma, one of the findings in thymoma-associated multiorgan autoimmunity (TAMA), was made.

The patient was scheduled to undergo 4 cycles of chemotherapy with lobaplatin (50 mg/m^2^ [70 mg]) and paclitaxel liposome (150 mg/m^2^ [210 mg]), as well as 32 Gy of local thoracic radiotherapy. She was also prescribed potent topical corticosteroids for the skin lesions and systemic steroids (intravenous methylprednisolone [40 mg/d]). After 2 cycles of chemotherapy, the right pleural tumor volume was significantly reduced and the levels of CA-125 (24.18 μ/mL; range 0–35) and CA15–3 (16.97 μ/mL; range 0–31.3) returned to normal ranges. However, her skin lesions worsened as large sheets of extreme desquamation developed and were accompanied by acropodium keratinization and erythroderma (Fig. 1B). Thereafter, we adopted a systemic long-acting glucocorticoid (intravenous dexamethasone [5 mg/d]) and the eruptions gradually subsided with hyperpigmentation (Fig. 1C). Unfortunately, the patient's lung and liver metastases worsened and she eventually died of multiple organ failure approximately 7 months after admission to the hospital.

## 3 Discussion

TAMA was first proposed by Wadhera et al^[4]^ and is defined as an autoinflammatory disorder of the liver, intestines, or skin, which resembles GVHD histopathologically around the premise of a malignant thymoma and in the absence of hematopoietic stem cell transplantation. There are 25 case reports of TAMA with cutaneous manifestations in the English and Chinese literature, including our case. Seventeen of the 25 cases identified the histologic subtype of thymoma per WHO classification—6 cases were classified as type B1 thymomas, 4 cases were reported to be type B2 and type B3 thymomas, 1 case was a type A thymoma, and 2 cases were type AB thymomas. In our case, the patient had a second biopsy from the right mediastinal pleura which demonstrated a type B3 thymoma that was different from the initial pathologic type (AB) on her initial thymoma diagnosis, while presenting with GVHD–like erythroderma. The pathologic types are similar to other thymoma-associated autoimmune diseases, such as MG, Good syndrome, and autoimmune cytopenia.^[5]^ Of the 25 patients with TAMA with cutaneous manifestation, 80.0% (20/25) resulted in a fatal course, which agrees with the current understanding that cutaneous manifestations of TAMA are associated with a poor prognosis.^[4,6,7]^

The eruption characteristics of TAMA can be heterogeneous in appearance, varying from confluent keratotic papules and scaly erythema, to morbilliform eruptions, erythroderma, and mucosal erosions. The differential diagnosis can be wide—including dermatitis herpetiformis, psoriasis, lichen planus, pityriasis rosea, lichenoid drug eruption, viral infections, paraneoplastic pemphigus, and persistent pruritic eruption of adult-onset Still disease.^[6–8]^ The most common pathologic findings of GVHD-like erythroderma are parakeratosis, necrotic keratinocytes, and interface and perivascular dermatitis. Only necrotic keratinocytes were thinly scattered in the epidermis, but the immunohistochemical evaluation showed infiltration of more CD8+ than CD4+ T cells and a marked decrease in CD1a+ Langerhans cells in the epidermis, which correspond to observed characteristics of TAMA in our case.^[6,7]^

Thymus is a primary lymphoid organ and plays an important role in immune regulation, including the differentiation and maturation of T-lymphocytes, positive and negative selection of T-cells, and maintenance of autoimmune tolerance. Risk for autoimmune diseases can be high in the setting of immune system disorders; however, the pathologic mechanism underlying a GVHD-like reaction in thymomas has not been fully illuminated. However, several theories exists, one of which is the “AIRE theory.”^[9,10]^ AIRE plays an important role in negative selection, which eliminates autoreactive T lymphocytes. A complete absence of AIRE in thymomas leads to autoreactive T-cell growth and their infiltration to the epidermis. The second theory is the “regulatory T-cell (Tregs) theory.” Tregs, especially natural Tregs, maintain peripheral tolerance and inhibit autoimmune responses by secreting bioactive factors with inhibitory effects. Liu et al^[9]^ observed the level of Foxp3, which is a specific marker for Tregs mRNA transcripts, was significantly lower in patients with thymoma-related autoimmune diseases. Hanafusa et al^[11]^ also reported that the percentage of skin-infiltrating FoxP3+ Tregs per number of CD4 T cells was decreased in patients with thymoma-associated multiorgan autoimmunity. Those findings suggest that Tregs deficiency plays a key role in TAMA.

The treatment for GVHD-like erythroderma has not been established; however, steroids (topical or systemic), narrow-band UVB phototherapy, and immunosuppressive drugs (cyclosporine or mycophenolate) are frequently used. The skin lesions improve momentarily in most cases, but are associated with a poor prognosis. People who have TAMA die within 1 year. The treatment of the underlying disease in patients with thymomas consists of surgical resection, chemotherapy, and radiotherapy. Complete resection of the thymoma may be the best treatment protocol, but most patients with TAMA are not suitable for surgery. In our case, chemotherapy exhibited good anti-thymoma activity with a combination of lobaplatin and paclitaxel liposome, while the cutaneous symptoms were aggravated and perhaps represented a side effect of chemotherapy. Moreover, long-acting dexamethasone has more efficacy than short-acting methylprednisolone for erythroderma, but the risk of developing serious infections along with application of steroids should be considered.

In conclusion, the diagnosis of TAMA with cutaneous symptoms should be confirmed by a dermatologist. Recurrent thymoma in patients with refractory skin eruptions should be screened. Early detection, diagnosis, and treatment can avoid metastasis of the thymoma. If thymoma is promptly diagnosed in patients with TAMA, thymectomy is an option that may prolong survival.

## Acknowledgment

We thank the staff of the Department of Central Laboratory and Oncology at The First Affiliated Hospital of Jinan University for providing valuable clinical support.
